# Preclinical and clinical evaluation of futibatinib in combination with binimetinib in patients with advanced cancer

**DOI:** 10.1007/s10637-026-01618-y

**Published:** 2026-05-18

**Authors:** Jordi Rodon, Bert O’Neil, Christopher Lim, Natraj R. Ammakkanavar, Carlos Torrado, Kazuaki Matsuoka, Hiroshi Hirai, Akihiro Miura, Bailey Anderson, Leah Jin, Nanae Hangai, Amanda Long, Volker Wacheck, Lee Rosen

**Affiliations:** 1 MD Anderson Cancer Center, Houston, TX USA; 2 Community Health Network, Indianapolis, IN USA; 3 Division of Hematology-Oncology, UCLA, Los Angeles, CA USA; 4 Taiho Pharmaceutical Co. Ltd, Chiyoda-Ku, Tokyo, Japan; 5 Taiho Oncology, Inc., Princeton, NJ USA

**Keywords:** Futibatinib, FGFR, Binimetinib, MEK, Non-small cell lung cancer

## Abstract

**Introduction:**

*KRAS-*mutant NSCLC resistance to MEK inhibitors (MEKi) is related to compensatory upregulation of fibroblast growth factor receptor (FGFR) signaling. Futibatinib is a potent, covalent FGFR1–4 inhibitor approved for locally advanced/metastatic cholangiocarcinoma with *FGFR2* fusions. We conducted a bench-to-bedside study evaluating futibatinib plus the MEKi binimetinib preclinically, followed by a Phase Ib trial of patients with advanced cancer.

**Methods:**

The effect of futibatinib ± MEKi on cell signaling and proliferation of *KRAS*mt NSCLC lines (A549, LU99) were assessed in vitro. In a Phase Ib study, patients with advanced cancer who had exhausted standard therapies received futibatinib QD plus binimetinib BID orally following a “3 + 3” dose-escalation design. Primary objective was to establish the recommended Phase 2 dose (RP2D) based on safety. Secondary endpoints included pharmacokinetics and anti-tumor activity.

**Results:**

In vitro, MEKi with futibatinib resulted in additive or synergistic anti-tumor activity in *KRAS*mt NSCLC lines. Twenty-three patients received futibatinib with binimetinib at 4 dose levels. The most prevalent adverse events were reversible retinopathies in 20 patients, including 2 DLTs and limiting dose escalation beyond futibatinib 16 mg QD plus binimetinib 15 mg BID (MTD). There were no relevant drug-drug interactions between binimetinib and futibatinib. One patient harboring an *FGFR2* fusion had a partial response. No RP2D could be defined as the MTD was below active dose levels of binimetinib.

**Conclusion:**

Despite additive anti-tumor activity for FGFR and MEK inhibition in KRASmt NSCLC lines, the trial was stopped after dose escalation, as no RP2D was identified for futibatinib/binimetinib due to reversible retinopathies.

Trial register number.

NCT04965818.

Trial registration date.

20-September-2021.

**Supplementary Information:**

The online version contains supplementary material available at 10.1007/s10637-026-01618-y.

## Introduction

*KRAS*-mutant (*KRAS*mt) tumors are present in 20–25% of White patients with lung adenocarcinoma [1]. Although the KRAS inhibitors sotorasib and adagrasib represent an advance for selected *KRAS*mt non-small cell lung cancer (NSCLC) patients, both drugs demonstrated a median progression-free survival (PFS) of < 6 months in Phase III trials [2]. Thus, there continues to be an unmet need for patients with NSCLC and other non-NSCLC tumours harboring *KRAS*mt.

The mitogen‑activated protein kinase (MAPK) pathway regulates cell proliferation and differentiation, and is a key effector pathway for *KRAS* [3]. Pharmacologic inhibition of this pathway via MEK inhibition has shown limited efficacy in patients with *KRAS*mt NSCLC [4]. For example, a Phase II trial of the MEK inhibitor (MEKi) selumetinib demonstrated efficacy comparable to pemetrexed in advanced NSCLC [5]. The limited efficacy of MEKi may result from compensatory activation of redundant signaling pathways, including the fibroblast growth factor receptor 1 (FGFR1) signaling pathway, and feedback activation of other upstream receptor tyrosine kinases [1, 6]. Preclinical studies have shown that combining FGFR1 inhibition with MEKi enhances tumor cell death in *KRAS*mt cancer cells by disrupting FRS2-mediated signaling rebound [1, 6]. Additionally, targeting FGFR1 or FRS2, but not FGFR2/3, sensitized *KRAS*mt lung cancer cells to MEKi trametinib, with sensitivity correlating with the degree of phosphorylated FRS2 [1, 6]. Feedback reactivation of ERK signaling was not prevented using inhibitors targeting other kinases known to be reactivated by MAPK inhibition [1, 6]. Together, these findings provide a strong rationale for evaluating combined MEKi and FGFR inhibitors (FGFRi) as a novel and chemotherapy-free strategy to address the unmet need among patients with *KRAS*mt NSCLC and potentially other *KRAS*-driven cancers.

Futibatinib (TAS-120) is a novel, potent FGFRi that irreversibly blocks downstream FGFR1–4 signaling by covalent binding [7]. In a pivotal Phase II trial for patients with previously treated, unresectable or metastatic intrahepatic cholangiocarcinoma (iCCA) harboring *FGFR2* fusions or other rearrangements, futibatinib demonstrated clinically meaningful efficacy, with objective response rate (ORR) of 42% and median duration of response (DOR) of 9.7 months [7]. Common side effects included hyperphosphatemia (85%), alopecia (33.0%), dry mouth (30.1%), and diarrhea (28.2%) [7]. Based on this, futibatinib was approved in various geographies for patients with *FGFR2* fusion/rearrangement–positive iCCA. Futibatinib has also shown clinical activity across other tumor types with *FGFR1*–*3* alterations, including gastric, urothelial, and breast cancer [8].

Here, we report results from a bench-to-bedside study exploring anti-tumor activity of futibatinib in combination with different MEKi (trametinib, binimetinib, cobimetinib) in *KRAS*mt NSCLC cell lines, followed by a Phase Ib dose-escalation trial of futibatinib plus binimetinib in advanced cancer patients, evaluating safety, pharmacokinetics (PK), and preliminary antitumor activity.

## Methods

### FGFR signaling and proliferation assays

Human *KRAS*mt lung carcinoma cell lines (A549 [ATCC, Manassas, VA, US], LU99 [Japanese Cancer Research Resources Bank, Osaka, Honshu, Japan]) were exposed to futibatinib alone, a MEKi (trametinib, binimetinib, or cobimetinib) alone, or both. Drug concentrations ranged from 0.3 to 300 nmol/L. Three independent experiments were performed sequentially. Cell growth inhibition was analyzed using the Bliss independence model [9].

For signaling analysis, A549 cells were exposed to selumetinib (100 or 1000 nmol/L) or trametinib (10 or 30 nmol/L) for 7 days, followed by futibatinib (100 nmol/L). Protein extraction and analysis are provided in Supplementary Methods.

### Phase Ib clinical trial

#### Clinical trial design and patients

This open-label Phase Ib clinical trial (NCT04965818) was designed to evaluate safety and tolerability of futibatinib plus binimetinib at multiple doses to identify a recommended Phase II dose (RP2D) and dosing schedule. Dose escalation (part 1) followed a standard “3 + 3” design in adult patients with advanced cancers who had exhausted standard therapies. Dose expansion (part 2) was planned for *KRAS*mt NSCLC patients. Secondary objectives included safety, anti-tumor activity, and PK of futibatinib, binimetinib, and AR00426032 (the active metabolite of binimetinib). Further details are provided in Supplementary Methods.

Eligible patients were ≥ 18 years old, had Eastern Cooperative Oncology Group performance status (ECOG PS) of 0/1, adequate organ function, and advanced malignancies without remaining standard treatment options. Availability of tumor samples and confirmed *KRAS*mt status was required for part 2 only. The study was conducted in accordance with the Declaration of Helsinki and Good Clinical Practice guidelines of the International Council for Harmonisation [10, 11], with institutional review board approval and written informed consent from all patients.

#### Treatment

Patients received oral futibatinib once daily (QD) and binimetinib twice daily (BID) according to approved schedule on a 21-day cycle until documentation of progressive disease, unacceptable toxicity, or other discontinuation criteria. Initial dosing was one dose level (DL) below the approved monotherapy doses of futibatinib and binimetinib. The protocol was amended to allow a 1-week lead-in of futibatinib or binimetinib to better characterize overlapping adverse events (AEs). Two futibatinib doses (16 and 20 mg) and up to three different binimetinib doses (15, 30, and 45 mg) were planned.

#### Endpoints and assessments

The primary objective of Phase Ib was to determine the RP2D based on safety, including AEs and dose-limiting toxicities (DLTs) during cycle 1 (C1). Patients who received at least 75% of both planned study treatments were evaluable for DLT. Given known ocular side effects associated with FGFRi and MEKi, comprehensive ophthalmological exams, including optical coherence tomography [OCT], were performed at baseline, every 3 cycles thereafter and as clinically indicated; further details in Supplementary Methods. AEs and laboratory parameters were graded per CTCAE v5.0. Further secondary objectives included anti-tumor activity according to RECIST v1.1 [12] and PK of futibatinib and binimetinib.

#### PK analysis and bioanalytical methods

Plasma PK parameters for futibatinib, binimetinib, and AR00426032 were derived using non-compartmental analysis (Phoenix WinNonlin™ software, Certara, Radnor, PA, USA) at steady state conditions. Further analysis is described in Supplementary Methods. Bioanalytic methods for futibatinib concentrations have been described previously [13].

#### Statistical Analysis

Safety, anti-tumor activity, and PK analyses included all patients receiving at least 1 dose of study treatment. ORR and confidence intervals were calculated and two-sided 95% CI estimated using Clopper-Pearson method. Kaplan–Meier method was used to estimate median PFS, and Brookmeyer and Crowley method to calculate 95% confidence intervals (CIs). Analyses were performed using SAS® version 9.3 or higher (SAS Institute, Inc., Cary, NC, USA).

## Results

### Futibatinib plus MEKi on KRASmt NSCLC cell lines

**Fig. 1:**
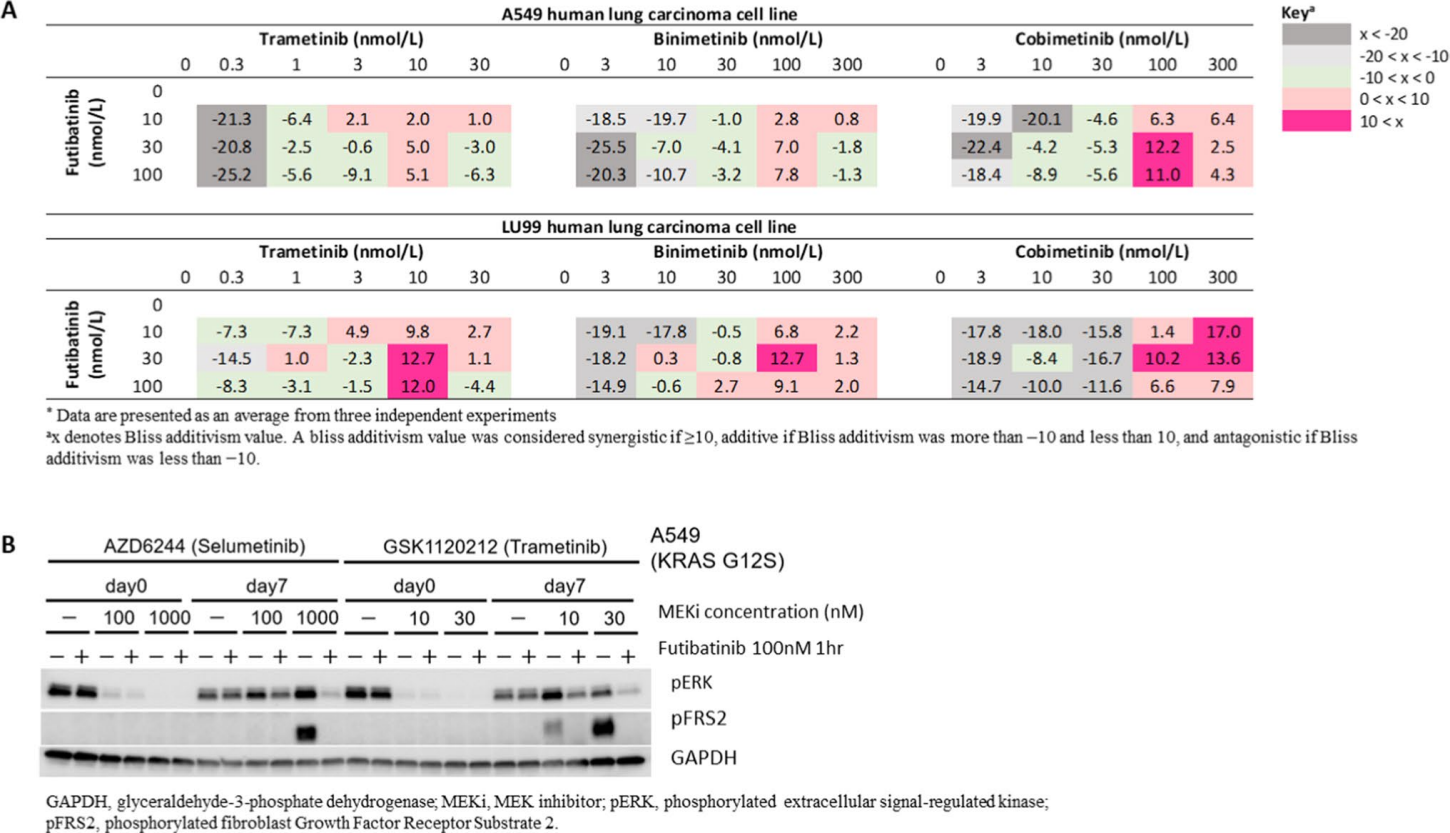
(A) Effect of futibatinib in combination with trametinib, binimetinib or cobimetinib on A549 and LU99 cell growth inhibition.* (B) Inhibition of pERK re-activation by futibatinib

In vitro, FGFR phosphorylation by futibatinib was suppressed in a dose-dependent manner (data not shown). Exposure of the *KRAS*mt human lung carcinoma cell line A549 to increasing concentrations of different MEKi (selumetinib, trametinib) led to a dose-dependent complete inhibition of pERK on day 0, whereas prolonged exposure for 7 days resulted in incomplete pERK inhibition only (Fig. 1b). FGFR signal analysis assay showed a clear rebound effect of FGFR signaling by upreg﻿ulation﻿ of FRS2 phosphorylation after 7 days of selumetinib or trametinib (Fig. 1b). However, when combined with futibatinib at nanomolar concentrations, we observed decreased MEKi induced FRS2 phosphorylation and re-activation of the MEK-ERK signaling was reduced, as demonstrated by inhibition of phosphorylated ERK (Fig. 1b).

To further assess the biological relevance of concurrent inhibition by MEKi and FGFRi, futibatinib was combined with binimetinib, trametinib, or cobimetinib at nanomolar concentrations for cell growth inhibition analysis of *KRAS*mt human lung carcinoma cell lines (LU99, A549). At most concentrations, futibatinib with binimetinib or trametinib showed additive or synergistic effects in LU99 cells and additive effects in A549 cells (Fig. 1A).

Together, these data demonstrate that futibatinib with different MEKi, effectively abrogates MEKi-induced FRS2 phosphorylation. This inhibition prevents subsequent re-activation of MEK-ERK signaling, resulting in additive anti-proliferative effects of futibatinib plus MEKi in *KRAS*mt human lung carcinoma cells. For clinical evaluation, binimetinib was selected for combination treatment with futibatinib in the Phase 1b trial.

### Phase 1b clinical trial

#### Patients

At data cut-off (July 24, 2023), 23 patients were enrolled in part 1 and received futibatinib with binimetinib. Baseline characteristics are summarized in Table 1. All patients had heavily pre-treated metastatic disease; 1 patient had NSCLC with unknown *KRAS*mt status. Most patients were male (56.5%), White (91.3%), had a median age of 60 years (range: 39–70), and an ECOG PS of 1 (78.3%). All patients had received prior systemic anticancer therapy, with a median of 4 prior lines of therapy (range: 1–7).

**Table 1:** Baseline characteristics

	*DL-2 (n* = *4) Futi 12 mg* + *bini 15 mg*	*DL-1 (n* = *10) Futi 16 mg* + *bini 15 mg with lead-in*	DL0 *(n* = 6) Futi 16 mg + bini 30 mg with lead-in	DL1 *(n* = 3) Futi 16 mg + bini 30 mg	*Total (N* = *23)*
Median age, years (range)	62.5 (57–65)	62.0 (43–70)	54.0 (39–65)	59.0 (57–60)	60.0 (39–70)
Sex, *n* (%)					
Male	2 (50.0)	7 (70.0)	4 (66.7)	0	13 (56.5)
Female	2 (50.0)	3 (30.0)	2 (33.3)	3 (100)	10 (43.5)
Race, *n* (%)					
White	3 (75.0)	9 (90.0)	6 (100)	3 (100)	21 (91.3)
Asian	0	1 (10.0)	0	0	1 (4.3)
Black/African American	1 (25.0)	0	0	0	1 (4.3)
Tumor type, *n* (%)					
Brain	0	2 (20.0)	0	0	2 (8.7)
Colon	1 (25.0)	2 (20.0)	4 (66.7)	0	7 (30.4)
Endometrium	0	0	0	1 (33.3)	1 (4.3)
GEJ	0	0	1 (16.7)	0	1 (4.3)
NSCLC	0	1 (10.0)	0	0	1 (4.3)
Ovary	0	0	0	1 (33.3)	1 (4.3)
Pancreas	1 (25.0)	0	1 (16.7)	0	2 (8.7)
Rectum	1 (25.0)	3 (30.0)	0	0	4 (17.4)
Skin	0	1 (10.0)	0	0	1 (4.3)
Other^a^	1 (25.0)	1 (10.0)	0	1 (33.3)	3 (13.0)
ECOG PS, *n* (%)					
0	2 (50.0)	1 (10.0)	1 (16.7)	1 (33.3)	5 (21.7)
1	2 (50.0)	9 (90.0)	5 (83.3)	2 (66.7)	18 (78.3)
Median (range) number of prior lines of systemic therapy	3.0 (1–5)	4.0 (1–7)	3.5 (2–7)	4.0 (4–7)	4.0 (1–7)

^a^Other tumor types were metastatic mucinous adenocarcinoma (n = 1), adenocarcinoma (n = 1), and adenoid cystic carcinoma (n = 1) BID, twice daily; DL, dose level; ECOG PS, Eastern Cooperative Oncology Group Performance Status; GEJ, gastroesophageal junction; NSCLC, non−small cell lung cancer; QD, once daily

#### Safety

The planned starting doses were futibatinib 16 mg QD and binimetinib 30 mg BID (DL1, N = 3). At this DL, all three patients developed blurred vision or retinopathy (Grade 1–2) within 1 week after initiation of concurrent futibatinib plus binimetinib. No retinal pigment epithelium detachment (RPED) was observed but ophthalmological exams by OCT showed fluid accumulation in subretinal layers. Study treatment was interrupted in all 3 patients and symptoms of blurred vision disappeared after 9–17 days without any further supportive treatment. One patient experienced re-occurrence of Grade 2 blurred vision after re-starting treatment. Other common treatment-related AEs (TRAEs) observed in at least 2 of the initial 3 patients included hyperphosphatemia, fatigue, and dry mouth (all Grade 1–2). There were no Grade ≥ 3 TRAEs (Table 2**, Supplementary Table 1**). Although reversible and not meeting the protocol-defined criteria of DLTs, this DL was deemed not tolerable by the sponsor and investigators due to the retinopathies observed.

**Table 2:** TRAEs reported in > 10% of all patients

Patients, *n* (%)	**DL-2** (*n* = 4) Futi 12 mg + Bini 15 mg		***DL-1 (n* = *10) Futi16 mg* + *bini 15 mg with lead-in***		***DL1 (n* = *3) Futi 16 mg* + *bini 30 mg***		***DL0 (n* = *6) Futi 16 mg* + *bini 30 mg with lead-in***		***Total (N* = *23)***	
	Any grade	Grade ≥ 3	Any grade	Grade ≥ 3	Any grade	Grade ≥ 3	Any Grade	Grade ≥ 3	Any grade	Grade ≥ 3
≥ 1 TRAE	4 (100)	0	10 (100)	6 (60.0)	3 (100)	0	6 (100)	3 (50.0)	23 (100)	9 (39.1)
Hyperphosphatemia	3 (75.0)	0	8 (80.0)	0	2 (66.7)	0	6 (100)	0	19 (82.6)	0
Retinopathy	3 (75.0)	0	5 (50.0)	0	2 (66.7)	0	4 (66.7)	1 (16.7)	14 (60.9)	1 (4.3)
Diarrhea	2 (50.0)	0	4 (40.0)	2 (20.0)	1 (33.3)	0	4 (66.7)	1 (16.7)	11 (47.8)	3 (13.0)
Fatigue	1 (25.0)	0	6 (60.0)	1 (10.0)	1 (33.3)	0	2 (33.3)	0	10 (43.5)	1 (4.3)
Increased AST	2 (50.0)	0	4 (40.0)	1 (10.0)	1 (33.3)	0	2 (33.3)	0	9 (39.1)	1 (4.3)
Blurred vision	2 (50.0)	0	3 (30.0)	1 (10.0)	2 (66.7)	0	0	0	7 (30.4)	1 (4.3)
Increased ALT	1 (25.0)	0	3 (30.0)	1 (10.0)	1 (33.3)	0	2 (33.3)	1 (16.7)	7 (30.4)	2 (8.7)
Rash	0	0	5 (50.0)	0	0	0	2 (33.3)	0	7 (30.4)	0
Constipation	1 (25.0)	0	2 (20.0)	0	1 (33.3)	0	1 (16.7)	0	5 (21.7)	0
Nausea	0	0	3 (30.0)	0	1 (33.3)	0	1 (16.7)	0	5 (21.7)	0
Gastroesophageal reflux disease	0	0	2 (20.0)	0	1 (33.3)	0	1 (16.7)	0	4 (17.4)	0
Stomatitis	0	0	2 (20.0)	1 (10.0)	1 (33.3)	0	1 (16.7)	0	4 (17.4)	1 (4.3)
Vomiting	0	0	3 (30.0)	0	0	0	1 (16.7)	0	4 (17.4)	0
Decreased appetite	1 (25.0)	0	0	0	0	0	2 (33.3)	0	3 (13.0)	0
Increased blood CPK	1 (25.0)	0	1 (10.0)	1 (10.0)	0	0	1 (16.7)	0	3 (13.0)	1 (4.3)
Increased blood creatinine	0	0	1 (10.0)	0	1 (33.3)	0	1 (16.7)	0	3 (13.0)	0

Includes events reported between first dose of either study drug and 30 days after last dose of either study drug. If a patient had two or more adverse events in the same System Organ Class (or with the same Preferred Term) with different CTCAE grades, then the event with the highest grade was used for that patient

*ALT alanine aminotransferase, AST aspartate aminotransferase, BID twice daily, CPK creatine phosphokinase, CTCAE Common Terminology Criteria for Adverse Events, QD once daily, TRAE treatment-related adverse event*

Consequently, lower combination doses were explored per protocol by de-escalating futibatinib to 12 mg QD and binimetinib to 15 mg BID (DL − 2, N = 4). This resulted in blurred vision (Grade 1–2) in 2 of the 4 patients after 2–9 days of treatment (Table 3). Treatment was interrupted in 1 of 2 patients with blurred vision, whereas symptoms of blurred vision in the other patient improved over time without stopping treatment. There were no Grade ≥ 3 treatment-emergent AEs (TEAEs; **Supplementary Table 2**) and no patients discontinued treatment due to TEAEs.

**Table 3:** Treatment-related ocular disorders reported across all dosing regimens

Patients, n (%)	DL-2 (*n* = 4) *Futi 12 mg + Bini 15 mg*^a^		^*DL−1 (n* = *10) Futi16 mg* + *bini 15 mg with lead−in*^b		^*DL1 (n* = *3) Futi 16 mg* + *bini 30 mg*^c		^*DL0 (n* = *6) Futi 16 mg* + *bini 30 mg with lead−in*^c		*Total (N* = *23)*	
	Any grade	Grade ≥ 3	Any grade	Grade ≥ 3	Any grade	Grade ≥ 3	Any grade	Grade ≥ 3	Any grade	Grade ≥ 3
Treatment-related ocular disorder	4 (100.0)	0	8 (80.0)	1 (10.0)	3 (100)	0	5 (83.3)	1 (16.7)	20 (87.0)	2 (8.7)
Retinopathy	3 (75.0)	0	5 (50.0)	0	2 (66.7)	0	4 (66.7)	1 (16.7)	14 (60.9)	1 (4.3)
Blurred vision	2 (50.0)	0	3 (30.0)	1 (10.0)	2 (66.7)	0	0	0	7 (30.4)	1 (4.3)
Subretinal fluid	0	0	0	0	1 (33.3)	0	1 (16.7)	0	2 (8.7)	0
Eye pain	0	0	0	0	0	0	1 (16.7)	0	1 (4.3)	0
Increased lacrimation	0	0	0	0	0	0	1 (16.7)	0	1 (4.3)	0
Eyelid swelling	0	0	0	0	0	0	1 (16.7)	0	1 (4.3)	0

If a patient had at least two adverse events in the same System Organ Class (or with the same Preferred Term) with different CTCAE grades, then the event with the highest grade was used for that patient

^a^Dosage of futibatinib and binimetinib at 60% and 33% of recommended dose for each study drug, respectively. ^b^Dosage of futibatinib and binimetinib at 80% and 33% of recommended dose for each study drug, respectively. ^c^Dosage of futibatinib and binimetinib at 80% and 67% of recommended dose for each study drug, respectively Includes events reported between first dose of either study drug and 30 days after last dose of either study drug

*BID* twice daily, *QD* once daily

Since toxicities were deemed manageable and reversible at lower doses, two additional cohorts of binimetinib 15 mg BID (N = 10; DL − 1); and futibatinib 16 mg QD plus binimetinib 30 mg BID (N = 6; DL1) were added. Both had a 7-day safety lead-in with binimetinib monotherapy before C1 to evaluate if early onset of retinopathies was due to single-agent binimetinib or combination with futibatinib, and to assess whether sequential initiation of futibatinib and binimetinib may be better tolerated with respect to ocular toxicities. Treatment with 7-day binimetinib monotherapy lead-in revealed similar TEAEs as observed in previous cohorts, with early onset of retinopathy in 9/16 patients, including 3/16 patients during binimetinib lead-in. Hyperphosphatemia, retinopathies with blurred vision, diarrhea, and fatigue remained the most common TRAEs in both cohorts (Table 2). One patient in DL − 1 experienced Grade 3 stomatitis meeting DLT criteria. At DL0, DLTs were observed in one patient with Grade 3 retinopathy (day 22) and 1 patient with Grade 2 retinopathy (day 18) that resulted in permanent discontinuation of binimetinib. Given the incidence of 2 DLTs in 6 patients enrolled at DL0 and 8/9 patients experiencing retinal disorder at binimetinib 30 mg BID plus futibatinib 16 mg QD (DL1 and DL0), dose escalation was ceased. There were no deaths related to trial treatment across all cohorts.

Overall exposure to study treatment was limited across cohorts. The median duration of exposure to futibatinib and binimetinib were 50.0 days (range 7–209) and 51.0 days (range 12–217), respectively (**Supplementary Table 3**). The longest median duration for both futibatinib and binimetinib was observed at the lowest DL evaluated (DL − 2), but median for either drug was below 12 weeks. Median relative dose intensity for futibatinib was 71.4% (range 15–100) and 64.9% (range 17–100) for binimetinib across cohorts, with median relative dose intensity exceeding 80% for both drugs only at the lowest DL evaluated (DL − 2) (**Supplementary Table 3**).

Nearly all patients (87%) required dose interruptions due to TEAEs across all DLs (**Supplementary Table 1**). TEAEs leading to futibatinib discontinuation were reported for 3 (13%) patients and for 4 (17.4%) patients due to binimetinib AEs. The primary reason for discontinuation of treatment was radiographic disease progression (52.2%).

Considering safety profiles and DLTs across the four cohorts, the MTD for the combination treatment was futibatinib 16 mg QD plus binimetinib 15 mg BID (DL − 1). There was no RP2D defined for the combination as the MTD was deemed to be below the DLs deemed to be required to achieve biological active DLs of binimetinib.

#### PK

Futibatinib with binimetinib PK profiles were assessed for potential drug-drug interactions (DDIs). Futibatinib PK profiles and exposure parameters were similar for cohorts receiving futibatinib 16 mg irrespective of binimetinib dose (**Supplementary Fig. 1**, **Supplementary Fig. 2)**. Mean binimetinib concentration increased with increasing dose. Binimetinib concentrations were within the variability of the historical data when administered as a single dose compared to when given in combination with futibatinib [14]. Comparable results were observed for binimetinib’s active metabolite, AR00426032.

#### Anti-tumor activity

Overall, the anti-tumor activity was limited with a confirmed ORR of 4.3% across all 23 patients enrolled. One partial response (PR) was observed in a patient with gastroesophageal junction cancer harboring an *FGFR* fusion treated with DL1. Time to response was 1.4 months and DOR was 4.4 months in this patient. The disease control rate was 39.1% and median PFS across all cohorts was 2.8 (95% CI, 1.6–4.5) months, with 3- and 6-month PFS rates of 38.5% (95% CI, 16.0–60.8) and 20.5% (95% CI, 4.2–45.5), respectively (Table 4).

**Table 4:** Summary of efficacy

	DL-2 (*n* = 4) Futi 12 mg + Bini 15 mg	*DL-1 (n* = *10) Futi16 mg* + *bini 15 mg with lead-in*	*DL1 (n* = *3) Futi 16 mg* + *bini 30 mg*	*DL0 (n* = *6) Futi 16 mg* + *bini 30 mg with lead-in*	*Total (N* = *23)*
Confirmed best overall response, *n* (%) CR PR SD PD NE/unknown^a^	0 0 2 (50.0) 1 (25.0) 1 (25.0)	0 0 4 (40.0) 3 (30.0) 3 (30.0)	0 0 1 (33.3) 0 2 (66.7)	0 1 (16.7) 1 (16.7) 4 (66.7) 0	0 1 (4.3) 8 (34.8) 8 (34.8) 6 (26.1)
ORR, % (95% CI)	0 (0.0–60.2)	0 (0.0–30.8)	0 (0.0–70.8)	16.7 (0.4–64.1)	4.3 (0.1–21.9)
PFS (months), median (95% CI)	2.8 (2.6–NE)	NE (1.3–NE)	4.0 (NE–NE)	1.7 (1.2–NE)	2.8 (1.6–4.5)
PFS rate, % (95% CI)					
At 3 months	33.3 (0.9–77.4)	53.6 (13.2–82.5)	100 (100–100)	16.7 (0.8–51.7)	38.5 (16.0–60.8)
At 6 months	0 (NE–NE)	53.6 (13.2–82.5)	0 (NE–NE)	NE (NE–NE)	20.5 (4.2–45.5)

^a^Five patients were not evaluable due to missing baseline and post-baseline tumor response assessments. One patient was not evaluable due to lack of post-baseline tumor response assessments. Exact 95% CIs of tumor response rate are two sided and calculated using the Clopper-Pearson method. PFS was calculated from the date of the first dose of either study drug to the date of first objective evidence of disease progression or date of death due to any cause, whichever occurs first. For a patient with none of these events, PFS was censored based on rules defined in the statistical analysis plan

*BID* twice daily, *CI* confidence interval, *CR* complete response, *NE* not evaluable, *ORR* objective response rate, *PD* progressive disease, *PFS* progression-free survival, *PR* partial response, *QD* once daily, *SD* stable disease

Due to limited anti-tumor activity, no further protocol planned efficacy endpoints were analyzed. Based on safety profile and limited anti-tumor activity, the trial was discontinued before initiation of the part 2 dose expansion in *KRAS*mt NSCLC.

## Discussion

Combination treatments with targeted agents are an important strategy in oncology to overcome resistance due to compensatory activation of alternative signaling pathways. In the current study, we first confirmed in vitro that MEK inhibition by different MEK-targeting tyrosine kinase inhibitors (TKIs) in *KRAS*mt NSCLC cell lines resulted in dose-dependent re-activation of MEK-ERK signaling via the FGFR signaling pathway. FGFR inhibition by futibatinib with different MEKi abrogated MEKi-induced phosphorylation of FRS2 and led to additive/synergistic antiproliferative effects in two *KRAS*mt NSCLC cell lines. Collectively, these non-clinical data support the rationale for investigating futibatinib with a MEKi in patients with advanced *KRAS*mt cancer.

However, combining an FGFRi and a MEKi led to tolerability issues. Specifically, the incidence of retinopathies exceeded previously reported rates for either futibatinib or binimetinib monotherapy. The historical rates of retinopathy reported are about 8% of patients receiving futibatinib and 20% receiving binimetinib plus encorafenib with events being generally reversible [15, 16]. Despite dose reductions below the approved dose of futibatinib monotherapy in combination with binimetinib, 16 (69.6%) patients experienced AEs of retinal disorders, including two of six patients who met DLT criteria (both DL0).

Retinopathies as a class of ocular toxicities are known on-target AEs of MEKi and FGFRi [17–19]. Both FGFR and MEK downstream signaling impact the MAPK pathway, which plays a crucial role in ocular development and involves signaling components expressed in the retina [20]. A systematic review and meta-analysis showed that MEKi, alone or in combination with other targeted therapies or chemotherapy, significantly increased risk of ocular AEs by 8.7%, versus treatment without MEKi [21]. In a Phase I trial of binimetinib in 93 patients with advanced solid tumors, MTD was reduced from 60 to 45 mg BID due to frequency of ocular toxicities at the higher dose [14]. Of 18 patients with ocular toxicities, median time-to-event onset after initiation of binimetinib was 17 days; ocular toxicities were primarily Grade 1/2 and managed with dose modifications [14]. Binimetinib is approved in the US and other countries in combination with encorafenib, a BRAF inhibitor, for *BRAF*-mutated metastatic melanoma based on results of the Phase III COLUMBUS trial, which reported retinopathy in 20% of patients with 6% requiring dose modifications [22]. Disease pathology affecting the eye has been also reported for FGFRi, with the ocular surface and retina most commonly involved, manifesting clinically as dry eye and retinopathy, respectively [23]. In a pooled safety analysis of 469 patients treated with futibatinib across three Phase I and II trials, futibatinib-related retinal disorders were reported in 38 patients (8.1%; all Grade 1/2), median time to onset was ~ 1 month (32.5 days) and all cases of Grade 2 retinal disorders resolved to Grade ≤ 1 over a median of 21 days following dose modifications [18]. Other treatment-related ocular disorders (any Grade) included dry eye, blurred vision, and cataract [18]. Ocular toxicities were manageable and most cases resolved quickly with dose modification, consistent with previous reports for FGFRi [18, 19].

In the current study, ocular toxicities were reported in 20 (87.0%) patients across all doses investigated. Most ocular disorders were manageable with dose reductions/interruptions and resolved within days, except in two patients. To determine whether observed retinopathy was due to combination treatment, a safety lead-in for single agent binimetinib or futibatinib was added to the study. Despite this, similar ocular AEs were observed and the incidence of retinopathy reported (60.9%) exceeds that previously reported for futibatinib monotherapy (retinal disorders, 8.1%) and for the combination of binimetinib with encorafenib (retinal disorders; 20%) [18, 22, 24, 25]. Although the retinopathies observed were manageable with treatment interruptions, there was no specific corrective therapy or patient selection criteria to manage or mitigate them.

Other AEs observed were consistent with the known safety profile for futibatinib and binimetinib and no additional new safety concerns were observed. Consistent with other futibatinib monotherapy studies[18], hyperphosphatemia was the most frequently observed TRAE (82.6%).

Preliminary anti-tumor activity was limited, with one patient achieving a PR and eight patients with stable disease. The patient who achieved a PR harbored an *FGFR2* fusion and may also have responded to futibatinib monotherapy. Notably, patients were heavily pretreated, having received a median 4 prior lines of therapy.

Exposure of futibatinib and binimetinib following co-administration was generally consistent with previously reported data for futibatinib or binimetinib monotherapy [14, 26], suggesting no clinically significant DDIs. Additionally, geometric mean accumulation ratios for binimetinib AUC (C1, day 1/lead-in) were < 2 and exposure of its active metabolite AR004260032 seemed unchanged following futibatinib treatment, also suggesting no relevant DDIs for the two compounds.

This trial has several limitations. While the in vitro data for futibatinib plus binimetinib support the crosstalk between the MEK and the FGFR signaling pathway as a resistance mechanism in *KRAS*mt NSCLC, there have been no in vivo experiments performed to confirm the study hypothesis due to the lack of readily available animal models. Still, the general concept of blocking RAS-MEK-ERK and FGFR signaling in vivo is known to result in additive activity in *KRAS*mt animal models under tolerated doses [1, 6]. For the clinical part, the dosing regimen used for combining futibatinib and binimetinib was based on the approved continuous dosing regimen for each drug. Alternative dosing strategies beyond the one-week lead-in—such as extended lead-in periods, intermittent dosing regimens, or pulsed MEK inhibition—were not evaluated and may have mitigated the ocular toxicities observed, which may stem from overlapping drug-related effects and/or the influence of intensive monitoring (including OCT). Nevertheless, different dosing schedules may attenuate therapeutic efficacy by providing only intermittent suppression of MEK/FGFR signaling in tumor cells, thereby enabling the emergence of resistant cell populations [27]. Additionally, patient selection in the dose escalation was not based on NSCLC patients with *KRAS*mt alterations, as planned for part 2 of the study. Although *KRAS*mt patients are not expected to exhibit a different safety or tolerability profile, potential early efficacy signals for futibatinib plus binimetinib in this population were unable to be assessed during the dose escalation part. Nonetheless, the trial generated valuable insights into overlapping on-target toxicities by co-targeting MEK and FGFR pathways, particularly ocular AEs, which may inform study design and mitigation strategies for future combination therapies.

In conclusion, reversible retinopathies requiring dose interruptions and precluding administration of futibatinib with binimetinib at DLs deemed to be required for biological activity led to early study termination after dose escalation. The data reported here underscore that targeting crosstalk between molecular pathways for improved anti-tumor activity may inadvertently lead to additive on-target toxicities that are intolerable for patients, as is seen with other drug combinations targeting other signaling pathways in different tumor types [28–30]. While the observed ocular toxicities in this study are consistent with on-target effects of concurrent FGFR and MEK pathway inhibition, it remains to be elucidated whether the tolerability issues observed could be overcome by other targeted agents with different drug properties (e.g. mutation specific TKIs). Thus, these findings should be interpreted as compound and/or regimen specific, and not as definitive evidence against the underlying molecular strategy of simultaneous targeting of MEK plus FGFR signaling. The results emphasize the importance of incorporating proactive strategies to manage overlapping or unforeseen toxicities when designing combination regimens involving targeted therapies.

## Supplementary Information

Below is the link to the electronic supplementary material.

Supplementary file1 (DOCX 360 KB)

Supplementary file2 (DOCX 362 KB)

## Data Availability

The data analyzed in this study are on file with Taiho Oncology, Inc. and Taiho Pharmaceutical Co., Ltd., and are not publicly available. Data are available from the authors upon reasonable request with the permission of Taiho. Other data generated in this analysis are available within the article.
