# Supplementary material for: Preclinical and clinical evaluation of futibatinib in combination with binimetinib in patients with advanced cancer
Source: Invest New Drugs. 2026 May 18;44(4):295–304. doi: 10.1007/s10637-026-01618-y (PMC13486011; doi:10.1007/s10637-026-01618-y)
Supplement: Supplementary file 1 [file 10637_2026_1618_MOESM1_ESM.docx]

Supplement to: **Preclinical and clinical evaluation of futibatinib in combination with binimetinib in patients with advanced cancer**

Jordi Rodon, Bert O’Neil, Christopher Lim, Natraj R Ammakkanavar, Carlos Torrado, Kazuaki Matsuoka, Hiroshi Hirai, Akihiro Miura, Bailey Anderson, Leah Jin, Nanae Hangai, Amanda Long, Volker Wacheck, Lee Rosen

Table of Contents

[Supplementary Methods	3](#_Toc209191851)

[Supplementary Table 1. Safety summary^*^	4](#_Toc209191852)

[Supplementary Table 2. TEAEs reported in > 10% of all patients	5](#_Toc209191853)

[Supplementary Table 3. Exposure of treatment across all dosing regimens	9](#_Toc209191854)

[Supplementary Fig. 1 Mean concentration-time profiles of A. binimetinib and B. AR00426032 on Cartesian (left, ± standard deviation) and semi-log (right) scales	10](#_Toc209191855)

[Supplementary Fig. 2 PK parameters for A. futibatinib, B. binimetinib, and C. AR00426032.	11](#_Toc209191856)

[Supplementary Appendix: Planned methods for phase 2	13](#_Toc209191857)

## Supplementary Methods

Key exclusion criteria included clinically significant toxicities (Common Terminology Criteria for Adverse Events [CTCAE] v5.0 grade ≥2) related to prior therapies, history of known phosphate homeostasis disorders, untreated brain metastases, and current evidence of clinically significant retinal or corneal disorders.

**Ophthalmological examination**

If patients had symptoms of new or worsening visual disturbances including diminished central vision, blurred vision, or loss of vision, a prompt ophthalmological examination is recommended. Abnormalities of the cornea and conjunctiva can usually be recognized via external ocular examination and routine slit lamp biomicroscopy. The retina is visible through fundoscopy after dilation of the pupil.

Ophthalmological examinations will be performed by an ophthalmologist or qualified delegate. Additional on-study evaluation may be performed as needed based on physician judgement and/or symptoms or signs of mineral deposits. In cases of retinal pigment epithelial detachment, events should be monitored at 2- to 3-week intervals.

Each evaluation will encompass:

- External ocular examination
- Routine slit lamp biomicroscopy of anterior ocular structures, including the anterior and posterior chambers (Fluorescein or rose Bengal or other dyes used to evaluate the ocular surface can be used according to institutional guidelines and local clinical practice)
- Dilation of the pupil with direct/indirect fundoscopy per institutional guidelines and local clinical practice
- Optical coherence tomography

## Supplementary Table 1. Safety summary^*^

| Patients, *n* (%) | **DL-2** (*n* = 4)  Futi 12 mg + Bini 15 mg | **DL-1** (*n* = 10) Futi16 mg + bini 15 mg  with lead-in | **DL1** (*n* = 3) Futi 16 mg + bini 30 mg | **DL0** (*n* = 6) Futi 16 mg + bini 30 mg  with lead-in | **Total** (*N* = 23) |
| --- | --- | --- | --- | --- | --- |
| Any TEAE | 4 (100) | 10 (100) | 3 (100) | 6 (100) | 23 (100) |
| Any TRAE | 4 (100) | 10 (100) | 3 (100) | 6 (100) | 23 (100) |
| SAE | 0 | 7 (70.0) | 1 (33.3) | 1 (16.7) | 9 (39.1) |
| Treatment-related SAE | 0 | 4 (40.0) | 0 | 1 (16.7) | 5 (21.7) |
| Grade ≥ 3 TEAE | 0 | 8 (80.0) | 1 (33.3) | 3 (50.0) | 12 (52.2) |
| Grade ≥ 3 TRAE | 0 | 6 (60.0) | 0 | 3 (50.0) | 9 (39.1) |
| TEAE leading to death | 0 | 0 | 0 | 0 | 0 |
| Dose modifications due to TEAEs |  |  |  |  |  |
| Futibatinib dose reduction | 0 | 4 (40.0) | 2 (66.7) | 4 (66.7) | 10 (43.5) |
| Futibatinib dose interruption | 3 (75.0) | 8 (80.0) | 3 (100) | 6 (100) | 20 (87.0) |
| Binimetinib dose reduction | 0 | 0 | 2 (66.7) | 4 (66.7) | 6 (26.1) |
| Binimetinib dose interruption | 3 (75.0) | 8 (80.0) | 3 (100) | 6 (100) | 20 (87.0) |
| TEAEs leading to futibatinib discontinuation | 0 | 2 (20.0) | 1 (33.3) | 0 | 3 (13.0) |
| TEAEs leading to binimetinib discontinuation | 0 | 2 (20.0) | 1 (33.3) | 1 (16.7) | 4 (17.4) |

*AE* adverse event, *BID* twice daily, *QD* once daily, *SAE* serious adverse event, *TEAE* treatment-emergent adverse event, *TRAE* treatment-related adverse event
^*^Includes all events reported between first dose of either study drug and 30 days after last dose of either study drug.

## **Supplementary Table 2**. TEAEs reported in > 10% of all patients

| Patients, *n* (%) |  | **DL-2** (*n* = 4)  Futi 12 mg + Bini 15 mg^a^ | | **DL-1** (*n* = 10) Futi16 mg + bini 15 mg  with lead-in^b^ | | **DL1** (*n* = 3) Futi 16 mg + bini 30 mg^c^ | | **DL0** (*n* = 6) Futi 16 mg + bini 30 mg  with lead-in^c^ | | **Total** (*N* = 23) | |
| --- | --- | --- | --- | --- | --- | --- | --- | --- | --- | --- | --- |
|  |  | Any grade | Grade ≥ 3 | Any grade | Grade ≥ 3 | Any grade | Grade ≥ 3 | Any grade | Grade ≥ 3 | Any grade | Grade ≥ 3 |
| ≥ 1 TEAE |  | 4 (100.0) | 0 | 10 (100.0) | 8 (80.0) | 3 (100.0) | 1 (33.3) | 6 (100.0) | 3 (50.0) | 23 (100) | 12 (52.5) |
| Hyperphosphatemia |  | 3 (75.0) | 0 | 8 (80.0) | 0 | 2 (66.7) | 0 | 6 (100.0) | 0 | 19 (82.6) | 0 |
| Retinopathy |  | 3 (75.0) | 0 | 5 (50.0) | 0 | 2 (66.7) | 0 | 4 (66.7) | 1 (16.7) | 14 (60.9) | 1 (4.3) |
| Diarrhea |  | 2 (50.0) | 0 | 4 (40.0) | 2 (20.0) | 1 (33.3) | 0 | 4 (66.7) | 1 (16.7) | 11 (47.8) | 3 (13.0) |
| Fatigue |  | 1 (25.0) | 0 | 6 (60.0) | 1 (10.0) | 2 (66.7) | 0 | 2 (33.3) | 0 | 11 (47.8) | 1 (4.3) |
| Increased AST |  | 2 (50.0) | 0 | 5 (50.0) | 2 (20.0) | 1 (33.3) | 0 | 2 (33.3) | 0 | 10 (43.5) | 2 (8.7) |
| Increased ALT |  | 1 (25.0) | 0 | 5 (50.0) | 2 (20.0) | 1 (33.3) | 0 | 2 (33.3) | 1 (16.7) | 9 (39.1) | 3 (13.0) |
| Blurred vision |  | 2 (50.0) | 0 | 3 (30.0) | 1 (10.0) | 2 (66.7) | 0 | 0 | 0 | 7 (30.4) | 1 (4.3) |
| Nausea |  | 0 | 0 | 5 (50.0) | 0 | 1 (33.3) | 0 | 1 (16.7) | 0 | 7 (30.4) | 0 |
| Rash |  | 0 | 0 | 5 (50.0) | 0 | 0 | 0 | 2 (33.3) | 0 | 7 (30.4) | 0 |
| Constipation |  | 1 (25.0) | 0 | 2 (20.0) | 0 | 1 (33.3) | 0 | 1 (16.7) | 0 | 5 (21.7) | 0 |
| Vomiting |  | 0 | 0 | 4 (40.0) | 0 | 0 | 0 | 1 (16.7) | 0 | 5 (21.7) | 0 |
| Gastroesophageal reflux disease |  | 0 | 0 | 2 (20.0) | 0 | 1 (33.3) | 0 | 1 (16.7) | 0 | 4 (17.4) | 0 |
| Dry mouth |  | 2 (50.0) | 0 | 0 | 0 | 2 (66.7) | 0 | 0 | 0 | 4 (17.4) | 0 |
| Stomatitis |  | 0 | 0 | 2 (20.0) | 1 (10.0) | 1 (33.3) | 0 | 1 (16.7) | 0 | 4 (17.4) | 1 (4.3) |
| Anemia |  | 0 | 0 | 0 | 0 | 1 (33.3) | 0 | 2 (33.3) | 0 | 3 (13.0) | 0 |
| Decreased appetite |  | 1 (25.0) | 0 | 0 | 0 | 0 | 0 | 2 (33.3) | 0 | 3 (13.0) | 0 |
| Decreased weight |  | 1 (25.0) | 0 | 1 (10.0) | 0 | 0 | 0 | 1 (16.7) | 0 | 3 (13.0) | 0 |
| Increased blood CPK |  | 1 (25.0) | 0 | 1 (10.0) | 1 (10.0) | 0 | 0 | 1 (16.7) | 0 | 3 (13.0) | 0 |
| Increased blood creatinine |  | 0 | 0 | 1 (10.0) | 0 | 1 (33.3) | 0 | 1 (16.7) | 0 | 3 (13.0) | 0 |

If a patient had at least two adverse events in the same System Organ Class (or with the same Preferred Term) with different CTCAE grades, then the event with the highest grade was used for that patient. Includes events reported between first dose of either study drug and 30 days after last dose of either study drug

^a^Dosage of futibatinib and binimetinib at 60% and 33% of recommended dose for each study drug, respectively. ^b^Dosage of futibatinib and binimetinib at 80% and 33% of recommended dose for each study drug, respectively. ^c^Dosage of futibatinib and binimetinib at 80% and 67% of recommended dose for each study drug, respectively

*ALT* alanine aminotransferase, *AST* aspartate aminotransferase, *CTCAE* Common Terminology Criteria for Adverse Events, *BID* twice daily, *CPK* creatine phosphokinase, *QD* once daily, *TEAE* treatment-emergent adverse event

## **Supplementary Table 3**. Exposure of treatment across all dosing regimens

|  |  | **DL-2** *(n =* 4) Futi 12 mg + bini 15 mg | **DL-1** *(n =* 10) Futi 16 mg + bini 15 mg  with lead-in | **DL0** *(n =* 6)  Futi 16 mg + bini 30 mg  with lead-in | **DL1** *(n =* 3)  Futi 16 mg + bini 30 mg | **Total** *(N =* 23) |
| --- | --- | --- | --- | --- | --- | --- |
| Duration of treatment (days), median (range) | |  |  |  |  |  |
| Futibatinib | | 82.0 (35–112) | 46.0 (7–129) | 72.0 (22–124) | 43.0 (34–209) | 50.0 (7–209) |
| Binimetinib | | 78.0 (35–112) | 49.0 (14–91) | 72.0 (12–124) | 50.0 (19–217) | 51.0 (12–217) |
|  | RDI (%), median (range) |  |  |  |  |  |
|  | Futibatinib | 85.0 (57–99) | 72.1 (48–100) | 27.8 (15–77) | 64.0 (33–90) | 71.4 (15–100) |
|  | Binimetinib | 86.8 (79–100) | 89.0 (57–100) | 36.8 (17–48) | 59.8 (50–91) | 64.9 (17–100) |

Duration of treatment (days) was calculated as date of the last dose – date of first dose + 1. Relative dose intensity (RDI) was calculated as cumulative dose divided by total dose planned. RDI is calculated within the treatment duration period. The total planned dose was calculated as the treatment duration multiplied by the initial planned daily dose.
*BID* twice daily, *QD* once daily, *RDI* relative dose intensity

Supplementary Fig. 1 Mean concentration-time profiles of A. binimetinib and B. AR00426032 on Cartesian (left, ± standard deviation) and semi-log (right) scales.

*Bini* binimetinib administered twice daily, *DL1* dose level 1, *DL2* dose level 2, *DL3* dose level 3, *DL4* dose level 4, *Futi* futibatinib administered once daily

**A	B**

## **Supplementary Fig. 2** PK parameters for A. futibatinib, B. binimetinib, and C. AR00426032.

*AUC_0–6_* area under the plasma concentration-time curve from time 0 to 6 hours, *AUC_inf_* area under the plasma concentration-time curve from time 0 to infinity, *C1D1* cycle1 day 1, *C_max_* maximum plasma concentration, *DL1* dose level 1 *DL2* dose level 2, *DL3* dose level 3, *DL4* dose level 4, *Futi* futibatinib administered once daily, *PK* pharmacokinetics, *T_max_* time to maximum plasma concentration

**A**

**B**

**C**

## Supplementary Appendix: Planned methods for phase 2

***Clinical trial design and patients***

Phase 2 (dose expansion) was planned to be an open-label, non-randomized, uncontrolled study of patients with KRAS-mutant non-small cell lung cancer who had progressed on prior standard of care therapy, including anti–programmed death (ligand) 1 therapy and platinum-based chemotherapy.

Approximately 18 patients were to receive futibatinib with binimetinib at the recommended phase II dose, as determined in part 1. The planned primary endpoint of the phase 2 portion was objective response rate (ORR) according to Response Evaluation Criteria in Solid Tumours v1.1 based on investigator assessment. Secondary endpoints were pharmacokinetics and preliminary anti-tumor activity (ORR [part 1 only], duration of response, and progression-free survival).
